# Effect of Age on Job Satisfaction and Emotional Exhaustion of Primary School Teachers in Greece

**DOI:** 10.3390/ejihpe10020047

**Published:** 2020-06-13

**Authors:** Sophia Anastasiou, Evaggelos Belios

**Affiliations:** 1 Faculty of Social Sciences, University of Ioannina, GR 45500 Ioannina, Greece; std096742@ac.eap.gr; 2 M.Ed. in Adult Education Course Program, Hellenic Open University, GR 26335 Patras, Greece

**Keywords:** human resources, burnout, job satisfaction, school management

## Abstract

The level of occupational burnout (OB) and job satisfaction (JS) was investigated in primary school teachers (n = 125) in the region of Epirus in Northwestern Greece. Teachers exhibited a high level of emotional exhaustion (EE), a medium level of depersonalization (DP), and a lack of personal accomplishment (PA). In our study, EE, which is a significant component of OB, varied according to intrinsic and extrinsic JS parameters. Teachers were less satisfied and more stressed with extrinsic job characteristics of their job, such as working conditions and working hours. Female teachers were more likely to exhibit increased satisfaction from intrinsic job characteristics, whereas male teachers were more likely to exhibit increased emotional exhaustion and lack of personal accomplishment. Job satisfaction had a significant negative impact on emotional exhaustion. Job satisfaction accounted (EE = 47.173 − 3.527*JS) for 35.1% of the total variation in the dependent variation of EE (F(1124) = 66.094, *p* < 0.001), indicating that job satisfaction had a significant negative effect on EE, such that an additional unit in job satisfaction will lower EE by 3.527. A Pearson correlation analysis revealed that age correlated negatively with emotional exhaustion (r = −0.204, *p* = 0.023). Proactive human resources policies may be required to protect the newly hired and less experienced teachers from exposure to stressful working conditions.

## 1. Introduction

Occupational burnout and job satisfaction are important and crucial parameters for successful human resource management for modern organizations. Job satisfaction refers to the attitude of employees toward various aspects of their work [1] and can have a positive effect on several parameters of their job, including productivity, efficiency, reduced absenteeism, staff turnover rate, and well-being in general (e.g., reduced risk for occupational burnout of employees) [2,3]. The significance of job satisfaction and occupational burnout has been reported in several professions. There is a plethora of evidence which suggest that satisfied employees are likely to exhibit increased productivity and more positive attitudes towards their assigned task [4,5,6,7,8].

Teachers’ job satisfaction is generally associated with different work features such as motivation, productivity, turnover rates, quality of work, and job efficiency. Teachers’ job satisfaction is a crucial parameter for both teaching efficiency and school performance. Satisfied teachers can be motivated and can work harder to achieve the set goals and objectives. On the contrary, dissatisfied teachers may exhibit increased levels of occupational stress and reduced performance [3,5,7,8,9].

Teachers’ commitment and satisfaction are affected by several extrinsic and intrinsic parameters, including personality traits, school leadership, working conditions, and social and economic factors. School leadership, emotional support, management feedback, and participation in decision making can improve the prospects of teachers’ job satisfaction [5,10].

Teachers tend to be satisfied more by the nature of their job and less by other parameters associated with work conditions and environment [11,12]. School leadership and organizational factors also contribute to teachers’ job satisfaction. School conflict, role ambiguity, work overload, bureaucracy, numerous law reforms, poor working environment, poor remuneration, low advancement prospects, and lack of adequate institutional management have been frequently reported as negative factors [13,14,15]. 

Job satisfaction depends on a wide range of variables, and it reflects the general attitude of an individual for their job, as well as how they view their profession, the working environment, and the wider general perspectives of the working environment. The overall level of job satisfaction reflects employees’ perceptions for the general job characteristics of their work (e.g., for teachers, they may enjoy working with children, inspiring students, and interacting with parents) as well the specifics of their work (e.g., their current school unit, current students, current school leader). A smaller but also significant effect of “social comfort” job characteristics can also contribute to shaping the overall level of job satisfaction. Wages, job security, and safety are considered as social comfort job characteristics that may affect job satisfaction [16]. For example, a teacher may like the nature of their job but may dislike some school parameters such as leadership, and may enjoy support from their fellow teachers or enjoy the feeling of social comfort from positive stimuli by the wider society for their job and the recognition of their job by the society [17]. 

The various components of job satisfaction can be grouped into intrinsic and extrinsic job characteristics. Intrinsic job satisfaction parameters are the attitudes of individuals towards their jobs, while extrinsic job satisfaction parameters entail the factors that relate to the environment at work [17]. Satisfaction refers to the positive feeling of a person to their job [18]. Therefore, the feelings are intrinsic if one looks at variables such as the job type, while extrinsic looks at the working condition such as supervisors, coworkers, and pay. Distinguishing between extrinsic and intrinsic elements in work contentment helps to determine the degrees of satisfaction [19].

Many factors influence work fulfillment of school teachers in Greece [5,9,10,11,12,20], and they can be categorized into some primary categories. The first category includes those factors that relate to the work settings, and the second category are factors associated with a particular job aspect. An effect of other factors was also reported for leadership, personality traits, experience, and age [5,9,10,11,12,20]. 

When considering gender as a personal factor, it is not easy to determine the difference between females and males concerning job satisfaction levels, considering no studies have found any significant difference. For instance, gender as a variable was a significant determinant only in the working condition aspect. As for age, experience, the status of marriage, education level, and the number of children, studies have reached contradictory conclusions on how they relate to work contentment. In the study conducted by Koustelios [20], about 40 schools were studied, of which 20 were primary schools. In this study, questionnaires were used to collect data, and the response rate was 49.2% of the 720 surveys. The instruments for evaluating job fulfillment included an Employee Satisfactory Inventory (ESI). The ESI measured six aspects of satisfaction: the job itself, conditions of work, organization, promotion, and pay. The five-point scale ranged from 1 to 5, with one being a strong disagreement and five being a strong agreement. Based on the results, the teachers seem to have a higher job satisfaction with the supervision and the job itself [20]. As for the pay and opportunities relating to promotion, teachers felt dissatisfied. While pay had the lowest variance, working conditions had the highest.

The significance of intrinsic job characteristics of teachers in Greece was also observed in other studies. For instance, Saiti and Papadopoulos [14] conducted a study in 2013 that was exclusively on primary school teachers in Attiki in Athens, Greece. It was found that among most teachers, there was more satisfaction with the nature of teaching, their colleagues, and the administration aspects. The satisfaction levels were low when it came to potential rewards, benefits, and salary. The study also concluded that gender is a predictor in the colleagues and in promotion aspects, while age was a predictor of the nature of work, colleagues, potential rewards, and administration. Similar results have been reported from other regions of Greece, which also observed that teachers exhibited an increased level of satisfaction from the intrinsic parameters of their profession [5,12]. 

Teachers in other European countries may exhibit differences in the sources of job satisfaction/dissatisfaction, according to the European survey TALIS in 2013. Spain was one of the participating countries with over 3000 teachers and 192 schools as participants [21]. The TALIS data were collected through questionnaires. According to the study, individual characteristics were found to be essential in job satisfaction compared to school characteristics. When it comes to individual components, the one factor with the most effect on job satisfaction is classroom discipline. As for school characteristics, the teacher–student relationship is the most important. Unfortunately, Greece was not included in the TALIS data analysis to facilitate a comparison. 

Primary school teachers face a carrier paved with challenges and rewards. In Greece, primary school teachers experience rapid changes in the curriculum as well as changes in the skills required for their job. For example, teachers in Greece faced the introduction of changes in the teaching methods; they also need to develop IT literacy skills and be trained to work with a multicultural student population as a result of the prolonged influx of economic migration and refugees in this country. In spite of the challenges, teachers have positive stimuli associated with their personal fulfillment as teachers and the recognition of their teaching profession by students, parents, and the society.

Occupational burnout is a significant parameter that can minimize employees’ effectiveness and productivity. Occupational burnout can occur when employees are exposed to unfavorable working conditions, and this condition can be assessed by investigating levels of emotional exhaustion (characterized by physical and psychological fatigue), depersonalization (characterized by cynical behavior and detachment from the job), and personal achievement (characterized by feeling inefficient/incompetent at work) of the employees [22,23]. Burnout is preceded by a lengthy exposure to occupational stress, which can eventually lead to burnout, and burnout is associated with depersonalization, reduced personal achievement, and emotional exhaustion [3,4]. Burnout problems can be seen in schools, with teachers who experience workload and psychological demanding work. The magnitude of the problem may increase by the length of the exposure to stressful job parameters and leads to occupational stress [3,5,12].

Occupational burnout may require a lengthy exposure to unfavorable conditions of the employees, and the first signs of this issue may be related to physical and psychological issues related to work and can gradually progress to emotional exhaustion, depersonalization, and a low sense of professional accomplishment. As a result, employees may enter the “professional burnout zone” and exhibit reduced productivity and low levels of job satisfaction [5,24,25,26]. Teachers are frequently exposed to demanding and stressful working conditions, and burnout is frequently reported globally [5,27]. Burnout can reduce employees’ performance, and the problem is manifested and established after prolonged exposure to unfavorable working conditions, which can result in occupational stress [25,28,29]. Although job satisfaction and occupational stress are not directly related, in practice, satisfied employees may be less stressed and thus work more efficiently. This has a positive long-term effect for the employees and their employer or their organization [30].

As discussed above, job satisfaction depends on a range of variables that may change during the teaching career of an individual. Teachers may have different aspirations and needs according to their career stage, professional growth, or personal needs. External variables may also change with time according to wider changes in the society. For example, in times of financial crisis, employees may face salary cuts and feel unsecure for the future. As a result of the prolonged economic problems of the Greek government, teachers together with other professions in the country had to perform their tasks with limited resources, reduced wages, and shortages in staff. These working conditions can lead to emotional resource depletion and be a source of job stress and emotional exhaustion [31].

Burnout and job satisfaction can interact with each other and can also be affected by several job characteristics, working conditions, organizational factors, personality traits, age, gender, and work experience [32,33]. As a result of a prolonged economic crisis and austerity measures in Greece, teachers have experienced rapidly changing working conditions that included school closures, relocation of teachers, aging workforce, and wage reductions. These changes may have an impact on teachers’ job satisfaction and burnout levels [12,26,34,35,36], but the effect may be modulated by age and work experience [32,33,37,38]. For example, with age, teachers may develop social skills and experience that may help them to be more efficient, be satisfied with their job, and cope with job related stress [39,40].

### Objectives of the Current Study:

The aim of the present work is to investigate the level of job satisfaction and burnout of primary school teachers in Greece during a period of a prolonged economic crisis, and how age or work experience may be a predictor of emotional exhaustion (EE), depersonalization (DP), and personal accomplishment (PA).

## 2. Materials and Methods

The present research was carried out during March and April 2017, in primary school units in the region of Epirus in Northwestern Greece. The head teachers of randomly selected (n = 19) school units (primary education) were informed about the aim of the present work and were asked to collaborate. In total, 150 questionnaires were distributed, and 125 completed questionnaires were collected (return rate 83.33%). The number of completed questionnaires corresponds to about 12% of the total number of primary school teachers in the region.

The distributed questionnaire included questions about demographic data as well as Maslach’s Burnout Inventory as adopted for usage in the Greek language by Kantas and Vassilaki [41] and Kokkinos [42].

Job satisfaction was measured with the use of the Job Satisfaction Scale [43], which has been previously validated and used in Greece and has a good (α = 0.71–0.91) internal consistency [12,44]. Teachers were asked to indicate on a Likert response scale (from *extremely dissatisfied* to *extremely satisfied*) the extent to which they are satisfied or dissatisfied with each of the 15 given statements perceived as job characteristics that are either extrinsic (e.g., physical work conditions, working hours, salary relationship with coworkers, quality of supervision) or intrinsic (e.g., freedom to choose work method, job recognition, opportunities for promotion). Teachers were also asked, “*What is the most stressful factor of your job*?” The answers to this question were grouped in two groups, namely intrinsic and extrinsic job characteristics [45], and the percentage of teachers for each group was calculated. The teachers were grouped in five work experience groups (0–5 years, 6–10 years, 11–15 years, 16–20 years, and over 21 years) and four age groups (20–30 years old, 31–40 years old, 41–50 years old, and over 50 years old).

The data were analyzed using SPSS (version 14.01), and Pearson’s correlation was used to examine the relationships between the variables. Cronbach’s alpha coefficients were used to assess the internal consistency of the instruments. The Cronbach alpha coefficient for job satisfaction was 0.81, and it was 0.79 and 0.83 for extrinsic and intrinsic job characteristics, respectively, thus providing assurance for the internal consistency [46] of the data. A t-test was used to compare the scores of female and male teachers. A one-way MANOVA followed by univariate ANOVAs was used to assess the impact of age and work experience on the EE, DP, and PA of the participants. The null hypothesis of the test is that occupational burnout does not vary with age and work experience. 

## 3. Results 

The sample teachers who participated in the present work included 125 responders who completed the questionnaires, of which 62.4% were female and 37.6% were male. The participants were distributed in four age groups: 7.2% of the samples were 20–30 years old, 21.6% were 31–40 years old, 44.0% were 41–50 years old, and 27.2% were over 50 years old.

The professional experience was distributed in five age groups: 10.4% between 0–5 years, 8.6% between 6–10 years, 16.0% between 11–15 years, 36% between 16–20 years and 29.0% over 21 years.

### 3.1. Job Satisfaction and Dimensions of Burnout

The overall job satisfaction was 4.80 (±0.68), with similar values exhibited in intrinsic (4.74 ± 0.46) and extrinsic (4.62 ± 0.71) job characteristics (Table 1). There was no significant difference between female and male teachers in terms of the overall job satisfaction, but female teachers exhibited a higher level of job satisfaction from intrinsic job characteristics. Compared to male teachers, female teachers exhibited a lower score on EE and on a lower score on the lack of personal accomplishment (Table 1).

**Figure 1:**
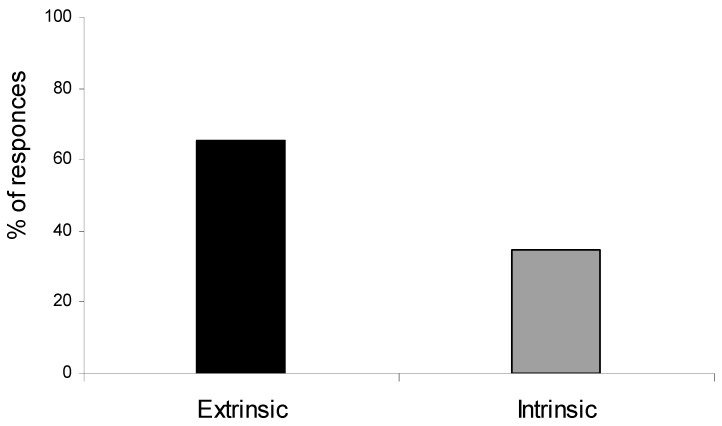
Primary school teachers’ perceptions on the sources of stress from extrinsic and intrinsic job characteristics. Out of the 15 job characteristics included in the job satisfaction survey, teachers were asked to select one as a source of stress.

The majority of the teachers who participated in the present work (59.2%) perceived the extrinsic job characteristics as the most important stressful parameters of their job, such as working conditions and erratic working hours (Figure 1).

The answers on the questions for the dimensions of burnout (Table 1) indicate that teachers exhibited a high score for emotional exhaustion, a medium score for depersonalization, and a high score for the lack of personal accomplishment. There was no gender effect on the overall job satisfaction but compared to male teachers, female teachers exhibited higher levels of satisfaction from intrinsic job characteristics and a lower level of EE, whereas male teachers exhibited a higher score for lack of personal accomplishment.

### 3.2. Correlation Analysis

The Pearson correlation analysis (Table 2) reveal that age correlated negatively with all other variables except working experience. However, only the relationship with working experience (r = 0.328, *p* < 0.001), job satisfaction (r = 0.001, *p* = 0.001), and emotional exhaustion (r = −0.204, *p* = 0.023) are statistically significant. 

Working experience correlated positively with age (r = 0.328, *p* < 0.001). Job satisfaction related negatively with EE (r = −0.593) and positively with age (r = 0.297, *p* = 0.001). A significant correlation between age and emotional exhaustion (r = −0.204, *p* = 0.023) was observed.

A MANOVA analysis (Table 3) was conducted to examine whether there were cross-group mean differences in occupational burnout based on categorical demographic variables. The *p* values of the Wilk’s Lambda for dependent variables EE and DP were 0.763 and 0.257, respectively (Table 3). Since the *p* values are greater than 0.05, the null hypothesis in regard to EE and DP failed to be rejected. Further, the *p* value of Wilk’s Lambda value of the variable PA was 0.03, which is less than 0.05. Since the *p* value is less than 0.05, the null hypothesis that there is no statistically significant difference in personal accomplishment based on respondents’ demographics was rejected. However, the univariate ANOVAs indicate a nonsignificant effect of both age (*p* = 0.147) and working experience (*p* = 0.131), and hence posthoc tests were not necessary. 

A regression analysis between each burnout component (EE, DP, and PA) as the dependent variable and job satisfaction (JS) was carried out. 

(i) Emotional exhaustion (EE)

The R^2^ for EE was 0.351, which suggests that the predictor job satisfaction accounts for 35.1% of the total variation in the dependent variation EE. Additionally, the predicted model is statistically significant (F(1,124) = 66.094, *p* < 0.001). The coefficient of job satisfaction is −3.527, and its *p* value is less than 0.05 (*p* < 0.001), indicating that job satisfaction has a significant negative effect on emotional exhaustion, such that an additional unit in job satisfaction will lower emotional exhaustion by 3.527. Therefore, the equation of the regression model will be EE = 47.173 − 3.527(JS).

(ii) Depersonalization (DP)

The R^2^ for depersonalization regressed against job satisfaction is 0.001, which suggests that job satisfaction accounted for only 0.1% of the variation in the outcome variable DP. However, the predicted model is statistically insignificant (F(1124) = 0.143, *p* = 0.706). This could be explained by the fact that the negative coefficient of job satisfaction of −0.027 is statistically insignificant since its *p* value is greater than 0.05 (*p* = 0.706).

(iii) Personal accomplishment (PA)

The value of the multiple of R when PA was regressed against job satisfaction is 0.004, which suggests that the predictor of job satisfaction accounts for 0.4% of the total variation in the dependent variation EE. Moreover, the predicted model is statistically insignificant (F(1124) = 0.446, *p* = 0.506). The coefficient of job satisfaction is −0.058 with a *p* value of 0.506, which is greater than 0.05. This indicates that the negative effect of job satisfaction on personal accomplishment is statistically insignificant. A significant negative effect of job satisfaction on emotional exhaustion is exhibited. There is no significant effect of job satisfaction on DP and PA. More research and a larger sample may be required to confirm this.

Based on the regression analysis, the results of the present work indicate that job satisfaction has a negative impact on emotional exhaustion. There was no gender effect on the overall job satisfaction, but compared to male teachers, female teachers exhibited higher levels of satisfaction from intrinsic job characteristics and a lower level of EE, whereas male teachers exhibited a greater score for lack of personal accomplishment. Moreover, there are no statistically significant differences in EE, DP, and PA based on respondents’ age and work experience. Specifically, there is no statistically significant difference in emotional exhaustion (F(240, 4) = 0.717, *p* = 0.763, Wilk’s sΔ = 0.0.001), depersonalization (F(46,198) = 1.149, *p* = 0.257, Wilk’s sΔ = 0.6230), and personal accomplishment (F(30, 112) = 1.637, *p* = 0.025, Wilk’s sΔ = 0.659) based on the respondents’ age. It is important to note that despite the *p* value of personal accomplishment being less than 0.05, the effect of age is statistically insignificant, indicating that personal accomplishment does not differ based on the age. 

## 4. Discussion

The significance of the extrinsic and intrinsic characteristics for job satisfaction has been demonstrated in several professions [45,47], including teachers [12,39,48]. The significance of extrinsic job characteristics (e.g., working conditions, wages) on the level of job satisfaction of teachers has been observed in other countries [3,49]; this was also observed in the present work, and extrinsic job characteristics were also a significant source of stress for the teachers who participated in the present work. Similar results have been reported from teachers working in public schools in Greece [5,12]. Teachers may enjoy the nature of their job, but extrinsic factors can have an impact on their job satisfaction. For example, teachers in public schools consistently exhibit lower levels of job satisfaction compared to their colleagues who work in private schools in Greece. This difference between the level of job satisfaction of teachers working in public and private schools stems from differences in working conditions, with private schools’ teachers exhibiting higher levels of job satisfaction from the support, infrastructure, and school management compared to those in public schools [39]. In addition to job satisfaction, burnout components may be affected according to working conditions. For example, as a result of austerity measures initiated during the prolonged economic crisis in Greece, teachers’ gross income has been drastically reduced. In the same manner, the entire public sector in Greece was downsized and operated with limited resources. School salaries were cut by 40%, and shortages in staff and lack of resources became a norm in the Greek public sector (12.These conditions can result in emotional resource depletion leading to emotional exhaustion [50], but the effect may vary according to personality traits and demographics. Nevertheless, a recently published study presented some evidence suggesting that teachers in Greece are exhibiting an alarmingly rising level of EE during the long period of economic crisis currently in this country and the unfavorable changes in some of the extrinsic job characteristics of their job [51].

In the present work, emotional exhaustion correlated with age, and female teachers exhibited lower levels of EE compared to male teachers. In addition, job satisfaction correlated positively with age and negatively with EE. In Greece, teachers are hired in public schools via a national hiring system and exams. The age may vary according to the periodicity in which the Greek government may organize the hiring process. It may also vary between different levels of education, skills, and subjects, with demand for computer teachers being higher compared to math teachers, for example.

Work experience may be negatively or positively correlated with the three dimensions (EE, DP, PA) of burnout. In some professions, length of service is associated with prolonged exposure to demanding and stressful working conditions. For example, the experience of special education teachers is a predictor for emotional exhaustion and depersonalization [47,48]. In other professions, the length of service may be negatively correlated with the level of burnout. For example, experienced employees may develop coping strategies [4,52,53] as well as social [39] and professional skills [40] or they may enjoy increased salaries and better working conditions, and all these factors may contribute to reducing the risk of occupational burnout of employees [50,51,52,53].

Irrespective of the gender, experienced and older primary school teachers may exhibit high scores of job satisfaction due to differences in their salary, working hours, responsibilities, and their perceptions for their efficacy, as compared to the less experienced teachers. Furthermore, in Greece, experienced primary education teachers may be more likely to be promoted, have reduced work load, be able to unofficially select their teaching classes, and be less exposed to stressful working conditions compared to less senior and less experienced teachers. Moreover, teaching experience and age may help some teachers to improve their teaching efficacy and emotional intelligence [18,39]. This potential beneficial effect of work experience is also reflected in the positive correlation between work experience and job satisfaction exhibited in the present work (Table 2). Furthermore, other factors may interact with job satisfaction and its characteristics. For example, a mediating effect of self-efficacy and school climate on the job satisfaction of primary school teachers has been reported [52,53,54].

The results of the present work indicate that proactive human resources policies may be required to protect the newly hired and less experienced teachers from burnout. Personality traits, age, experience, and working conditions can mediate the effect of job characteristics on job satisfaction [54,55,56,57,58,59,60,61]. In turn, unsatisfied teachers may gradually develop negative emotions for their job and be emotionally exhausted [62,63]. In the present work, a high level of emotional exhaustion was associated with reduced job satisfaction. Emotional exhaustion is a precursor to depersonalization, which subsequently can lead to lack of personal accomplishment [34,55]. Burnout can lead to decreased job performance, lack of enthusiasm and commitment, and reduced job satisfaction [55,56,57,58,59,60,61,62]. Educational policy makers could initiate mentoring or aiding initiatives that may be employed to assist teachers in developing skills. Leadership style can also contribute in reducing the level of exposure to stressful conditions of teachers [5,10,15,63]. For this reason, school leaders should be encouraged to exploit all available tools to handle the increased risk for occupational burnout of the younger or less experienced teachers.

## 5. Conclusions

The results of the present work indicate that female teachers were more likely to exhibit increased satisfaction from intrinsic job characteristics whereas male teachers were more likely to exhibit increased emotional exhaustion and lack of personal accomplishment. Frequent sources of stress were extrinsic job characteristics such as working conditions and working hours. Job satisfaction and age had a significant negative impact on emotional exhaustion. The results of the present work could be used by managers and policymakers for assessing and preventing the development of occupational burnout in their workforce.

## Figures and Tables

**Table 1:** Job satisfaction and occupational burnout of primary school teachers (n = 125) in Epirus in Northwestern Greece.

Variable	Mean Score (±SD)	Gender (Male/Female)	
*Job satisfaction*			
Overall job satisfaction (Min-max: 3.05–5.8)	4.80 (±0.68)	4.81 (±0.69)	4.79 (±0.65)
Intrinsic characteristics of job satisfaction (Min-max: 3.7–6.0)	4.74 (±0.46)	4.81 (±0.45)	4.66 * (±0.43)
Extrinsic characteristics of job satisfaction (Min-max: 3.2–5.8)	4.62 (±0.71)	4.70 (±0.58)	4.62 (±0.36)
*Components of Occupational Burnout*			
Emotional exhaustion (EE) (Min-max: 16.7–39)	32.5 (±1.25)	31.01 (±5.17)	31.63 * (±3.85)
Depersonalization (DP) (Min-max: 5.4–14.4)	10.32 (±1.90)	10.45 (±1.43)	10.18 (±1.90)
Personal accomplishment (PA) (Min-max: 35.12–44.70)	39.21 (±0.96)	39.00 (±1.43)	39.73 ** (±0.99)

Note: The number of asterisks indicates the significance of difference (*t*-test) between female and male teachers; * (*p* < 0.05); ** (*p* < 0.01).

**Table 2:** Correlation analysis.

		Age	Working Experience	Job Satisfaction	EE	DP	PA
**Age**	Pearson Correlation	1	0.328 **	0.297 **	−0.204 *	−0.118	−0.124
	Sig. (2-tailed)		0.000	0.001	0.023	0.190	0.172
	N	125	125	125	125	125	125
Work Exp	Pearson Correlation	0.328 **	1	0.150	−0.068	0.051	0.085
	Sig. (2-tailed)	0.000		0.097	0.456	0.573	0.352
Job sat	Pearson Correlation	0.297 **	0.150	1	−0.593 **	−0.034	−0.061
	Sig. (2-tailed)	0.001	0.097		0.000	0.706	0.506
EE	Pearson Correlation	−0.204 *	−0.068	−0.593 **	1	0.126	0.044
	Sig. (2-tailed)	0.023	0.456	0.000		0.162	0.630
DP	Pearson Correlation	−0.118	0.051	−0.034	0.126	1	0.140
	Sig. (2-tailed)	0.190	0.573	0.706	0.162		0.123
PA	Pearson Correlation	−0.124	0.085	−0.061	0.044	0.140	1
	Sig. (2-tailed)	0.172	0.352	0.506	0.630	0.123	

Note: The number of asterisks indicates the significance of the correlation (2-tailed); *(*p* < 0.05); ** (*p* < 0.01). (n = 125).

**Table 3:** One-way MANOVA results (conducted using EE, DP, and PA subscales as the dependent variables).

Effect		Value	F	Hypothesis df	Error df	Sig.
EE	Pillai’s Trace	1.952	1.016	240	6	0.564
	Wilks’ Lambda	0.001	0.717b	240	4	0.763
	Hotelling’s Trace	91.186	0.38	240	2	0.926
	Roy’s Largest Root	60.799	1.520c	120	3	0.42
DP	Pillai’s Trace	0.412	1.13	46	200	0.281
	Wilks’ Lambda	0.623	1.149b	46	198	0.257
	Hotelling’s Trace	0.548	1.167	46	196	0.235
	Roy’s Largest Root	0.408	1.774c	23	100	0.028
PA	Pillai’s Trace	0.372	1.632	30	214	0.026
	Wilks’ Lambda	0.659	1.637b	30	212	0.025
	Hotelling’s Trace	0.469	1.642	30	210	0.024
	Roy’s Largest Root	0.319	2.273c	15	107	0.008

## References

[1] Wang M., Hu C., Huang M., Xie Y., Zhu W. (2019). The effect of emotional clarity and attention to emotion on job satisfaction: A mediating role of emotion regulation among Chinese medical staff. Asian J. Soc. Psychol..

[2] Wang G., Lee P.D. (2009). Psychological empowerment and job satisfaction: An analysis of interactive effects. Group Organ. Manag..

[3] Skaalvik E.M., Skaalvik S. (2017). Still motivated to teach? A study of school context variables, stress and job satisfaction among teachers in senior high school. Soc. Psychol. Educ..

[4] Akgemci T., Demirsel M.T., Kara Ö. (2013). The effect of psychological resilience on employees’ burnout level. Acad. J. Interdiscip. Stud..

[5] Anastasiou S., Papakonstantinou G. (2014). Factors affecting job satisfaction, stress and work performance of secondary education teachers in Epirus, NW Greece. Int. J. Manag. Educ..

[6] Chevalier S., Fouquereau E., Bénichoux F., Colombat P. (2019). Beyond working conditions, psychosocial predictors of job satisfaction, and work engagement among French dentists and dental assistants. J. Appl. Biobehav. Res..

[7] Abuhashesh M., Al-Dmour R., Masa’deh R.E. (2019). Factors that affect employees job satisfaction and performance to increase customers’ satisfactions. J. Hum. Resour. Manag. Res..

[8] DiMaria C.H., Peroni C., Sarracino F. (2020). Happiness matters: Productivity gains from subjective well-being. J. Happiness Stud..

[9] Koutouzis M., Malliara K. (2017). Teachers’ job satisfaction: The effect of principal’s leadership and decision- making style. Int. J. Educ..

[10] Sarafidou J.O., Chatziioannidis G. (2013). Teacher participation in decision making and its impact on school and teachers. Int. J. Educ. Manag..

[11] Saiti A. (2007). Main factors of job satisfaction among primary school educators: Factor analysis of the Greek reality. Manag. Educ..

[12] Panagopoulos N., Anastasiou S., Goloni V. (2014). Professional burnout and job satisfaction among physical education teachers in Greece. J. Sci. Res. Rep..

[13] Soto M., Rojas O. (2019). Self-efficacy and job satisfaction as antecedents of citizenship behaviour in private schools. Int. J. Manag. Educ..

[14] Saiti A., Papadopoulos Y. (2015). School teachers’ job satisfaction and personal characteristics: A quantitative research study in Greece. Int. J. Educ. Manag..

[15] Yao X., Yao M., Zong X., Li Y., Li X., Guo F., Cui G. (2015). How School Climate Influences Teachers’ Emotional Exhaustion: The Mediating Role of Emotional Labor. Int. J. Environ. Res. Public Health.

[16] Lambert S.J. (1991). The combined effects of job and family characteristics on the job satisfaction, job involvement, and intrinsic motivation of men and women workers. J. Organ. Behav..

[17] Bektas C. (2017). Explanation of intrinsic and extrinsic job satisfaction via mirror model. Bus. Manag. Stud. Int. J..

[18] Singh B. (2016). Effect of emotional intelligence and gender on job satisfaction of primary school teacher. Eur. J. Educ. Res..

[19] Omar M.K., Self M.J., Cole K.M., Rashid A.M., Puad M.M. (2018). Job Satisfaction and Motivation to Teach: Predicting Intrinsic and Extrinsic Factors Towards Retaining Career-Switchers in The Teaching Profession. Int. J. Educ. Psychol. Couns..

[20] Koustelios A.D. (2001). Personal Characteristics and Job Satisfaction of Greek Teachers. Int. J. Educ. Manag..

[21] OECD (2014). Talis 2013 Results: An International Perspective on Teaching and Learning.

[22] Rautakivi T., Siriprasertchok R., Korng V. (2019). Extrinsic work motivation of urban secondary school teachers: A case study of public secondary schools and a model secondary school in Phnom Penh, Cambodia. Int. J. Manag. Educ..

[23] Ferradás M.D.M., Freire C., García-Bértoa A., Núñez J.C., Rodríguez S. (2019). Teacher profiles of psychological capital and their relationship with burnout. Sustainability.

[24] Sergiovanni T. (1967). Factors which affect satisfaction and dissatisfaction of teachers. J. Educ. Adm..

[25] Maslach C. (2003). Job burnout: New directions in research and intervention. Curr. Dir. Psychol. Sci..

[26] Portero de la Cruz S., Cebrino J., Herruzo J., Vaquero-Abellán M. (2020). A Multicenter Study into Burnout, Perceived Stress, Job Satisfaction, Coping Strategies, and General Health among Emergency Department Nursing Staff. J. Clin. Med..

[27] Desouky D., Allam H. (2017). Occupational stress, anxiety and depression among Egyptian teachers. J. Epidemiol. Glob. Health.

[28] Rasool S.F., Wang M., Zhang Y., Samma M. (2020). Sustainable work performance: The roles of workplace violence and occupational stress. Int. J. Environ. Res. Public Health.

[29] Schwarzer R., Schmitz G.S., Tang C. (2000). Teacher burnout in Hong Kong and Germany: A cross-cultural validation of the maslach burnout inventory. Anxiety Stress Coping.

[30] Halkos G., Bousinakis D. (2010). The effect of stress and satisfaction on productivity. Int. J. Product. Perform. Manag..

[31] Frenkel S.J., Li M., Restubog S.L.D. (2012). Management, organizational justice and emotional exhaustion among Chinese migrant workers: Evidence from two manufacturing firms. Br. J. Ind. Relat..

[32] Botou A., Mylonakou-Keke I., Kalouri O., Tsergas N. (2017). Primary school teachers’ resilience during the economic crisis in Greece. Psychology.

[33] Msuya O.W. (2016). Exploring Levels of Job Satisfaction among Teachers in Public Secondary Schools in Tanzania. Int. J. Educ. Adm. Policy Stud..

[34] Stagia D., Iordanidis I. (2014). Occupational stress and professional burnout of Greek teachers during a period of economic crisis (in Greek). Επιστημονική Επετηρίδα Παιδαγωγικού Τμήματος Νηπιαγωγών Πανεπιστημίου Ιωαννίνων.

[35] Kamtsios S., Lolis T. (2016). Do Greek teachers experience professional burnout? The role of demographic characteristics and daily stressful events (in Greek with an English Abstract). Επιστημονική Επετηρίδα Παιδαγωγικού Τμήματος Νηπιαγωγών Πανεπιστημίου Ιωαννίνων.

[36] Kamtsios S., Lolis T. (2016). Investigating burnout in Greek teachers: Are there any teachers at risk?. Hell. J. Psychol..

[37] Demirtas Z. (2010). Teacher’s job satisfaction levels. Soc. Behav. Sci..

[38] Simic Sasic S., Sorić I. (2010). Do personal characteristics of teachers contribute to the type of interaction they have with their students?. Drustvena Istrazivanja.

[39] Anastasiou S. (2020). The moderating effect of age on preschool teachers’ trait emotional intelligence in Greece and implications for preschool human resources management. Int. J. Educ. Pract..

[40] Antoniou A.S., Polychroni F., Vlachakis A.N. (2006). Gender and age differences in occupational stress and professional burnout between primary and high-school teachers in Greece. J. Manag. Psychol..

[41] Kantas A., Vassilaki E. (1997). Burnout in Greek teachers: Main findings and validity of the Maslach burnout inventory. Work Stress.

[42] Kokkinos C.M. (2007). Job stressors, personality and burnout in primary school teachers. Br. J. Educ. Psychol..

[43] Warr P., Cook J., Wall T. (1979). Scales for the measurement of some work attitudes and aspects of psychological well-being. J. Occup. Psychol..

[44] Anastasiou S., Karipoglou K., Nathanailides C. (2014). Participation in decision making, productivity and job satisfaction among managers of fish farms in Greece. Int. Bus. Res..

[45] Anastasiou S., Garametsi V. (2020). Perceived leadership style and job satisfaction of teachers in Public and Private Schools. Int. J. Manag. Educ..

[46] Nunally N.C. (1978). Psychometric Theory.

[47] Duli S. (2016). Years of work experience, an important predictor of burnout in special education. Am. Sci. Res. J. Eng. Technol. Sci..

[48] Jovanović V., Karić J., Mihajlović G., Džamonja-Ignjatović T., Hinić D. (2019). Work-related burnout syndrome in special education teachers working with children with developmental disorders–possible correlations with some socio-demographic aspects and assertiveness. Eur. J. Spec. Needs Educ..

[49] Gkliati A., Saiti A. (2016). Job satisfaction in the health care sector: Empirical evidence from medical care in Greece. Eur. J. Econ. Bus. Stud..

[50] Ismail A., Ismail Y., Ibrahim Z., Leng C., Kiong P. (2009). Relationship between pay level, pay structure and job commitment in Malaysian public community college: The mediation role of distributive justice. South East Asian J. Manag..

[51] Anastasiou S. (2020). Economic crisis, emotional exhaustion, depersonalization and personal accomplishment of teachers in Greece. Humanit. Soc. Sci. Lett..

[52] Shoji K., Cieslak R., Smoktunowicz E., Rogala A., Benight C.C., Luszczynska A. (2016). Associations between job burnout and self-efficacy: A meta-analysis. Anxiety Stress Coping.

[53] Van Droogenbroeck F., Spruyt B., Vanroelen C. (2014). Burnout among senior teachers: Investigating the role of workload and interpersonal relationships at work. Teach. Teach. Educ..

[54] Katsantonis I.G. (2020). Investigation of the Impact of School Climate and Teachers’ Self-Efficacy on Job Satisfaction: A Cross-Cultural Approach. Eur. J. Investig. Health Psychol. Educ..

[55] Zgliczyńska M., Zgliczyński S., Ciebiera M., Kosińska-Kaczyńska K. (2019). Occupational burnout syndrome in polish physicians: A systematic review. Int. J. Environ. Res. Public Health.

[56] Babakus E., Cravens D.W., Johnston M., Moncrief W.C. (1999). The role of emotional exhaustion in sales force attitude and behavior relationships. J. Acad. Mark. Sci..

[57] Ajlan H.A. (2019). Factors influencing teachers’ job satisfaction a case study of public secondry schools In Buraidh City Saudi Arabia. Adv. Soc. Sci. Res. J..

[58] Nwibere B.M. (2014). Interactive relationship between job involvement, job satisfaction, organisational citizenship behaviour, and organizational commitment in Nigerian universities. Int. J. Manag. Sustain..

[59] Tsigilis N., Zachopoulou E., Grammatikopoulos V. (2006). Job satisfaction and burnout among Greek early educators: A comparison between public and private sector employees. Educ. Res. Rev..

[60] Lim N., Kim E.K., Kim H., Yang E., Lee S.M. (2010). Individual and work-related factors influencing burnout of mental health professionals: A meta-analysis. J. Employ. Couns..

[61] Iwu C., Ezeuduji I., Iwu I., Ikebuaku K., Tengeh R. (2018). Achieving quality education by understanding teacher job satisfaction determinants. Soc. Sci..

[62] Zarei E., Ahmadi F., Sial M.S., Hwang J., Thu P.A., Usman S.M. (2019). Prevalence of burnout among primary health care staff and its predictors: A study in Iran. Int. J. Environ. Res. Public Health.

[63] Chandolia E., Anastasiou S. (2020). Leadership and Conflict Management Style Are Associated with the Effectiveness of School Conflict Management in the Region of Epirus, NW Greece. Eur. J. Investig. Health Psychol. Educ..

